# Syncope and bifascicular block in the absence of structural heart disease

**DOI:** 10.1038/s41598-020-65088-9

**Published:** 2020-05-18

**Authors:** Ricardo Rivera-López, Mercedes Cabrera-Ramos, Laura Jordán-Martinez, Juan Jimenez-Jaimez, Rosa Macias-Ruiz, Eduardo Aguilar-Alonso, Ricardo Rivera-Fernandez, Emilio Sanchez-Cantalejo, Luis Tercedor, Miguel Alvarez

**Affiliations:** 1 Cardiology Department, Hospital Universitario Virgen de las Nieves. Granada, Granada, Spain; 2 Granada Institute of Biohealth Research (Granada. Ibs), Granada, Spain; 3 Intensive Care Unit. Hospital Infanta Margarita, Cabra (Córdoba), Spain; 4 Intensive Care Unit, ComplejoHospitalario de Jaén, Jaén, Spain; 5 Andalusian School of Public Health, Granada, Spain; 6 Epidemiology and Public Health Network Biomedical Research Consortium (CIBERESP), Madrid, Spain

**Keywords:** Cardiology, Health care

## Abstract

The treatment of patients with bifascicular block (BFB) and syncope in the absence of structural heart disease (SHD) is not well defined. The objective of our study is to compare pacemaker empirical implantation with the use of electrophysiological studies (EPS). This is a prospective cohort study that included 77 patients with unexplained cardiogenic syncope and BFB without structural heart disease between 1997 and 2012. Two groups: 36 patients received empirical pacemakers (Group A) and 41 underwent EPS (Group B) to guide their treatment. The incidence of syncope recurrence and atrioventricular block was lower in group A. Mortality and complication rates were similar between both groups. Multivariate analysis demonstrated a higher number of events (combined endpoint) in group B. Our study shows that treatment according to EPS does not improve the results of a treatment strategy based on empirical pacemaker.

## Introduction

Management of patients with syncope of unknown origin and bifascicular block (BBF) without significant structural heart disease remains a matter of debate. According to current European guidelines, an electrophysiology study (EPS) should be performed as initial approach and an event recorder or permanent pacemaker implanted based upon the results^1,2^. However, this recommendation is not supported by large or prospective studies, and some authors postulate the potential benefit of a direct pacemaker implant giving the 40–50% reported incidence of atrioventricular (AV) block in the 2 years post-EPS^2–4^.

There is lack of prospective information regarding the clinical benefit of an empirical pacing approach compared to a treatment based on EPS results, in patients without structural heart disease.

Our aim is to compare the clinical outcome of patients with syncope of unknown etiology and BFB without significant structural heart disease treated empirically with permanent pacemaker implantation compared to those whose treatment approach was based on the results of an EPS.

## Methods

### Patients and treatment algorithm

We designed a cohort study with prospective follow-up to include patients presenting with unexplained syncope and BFB defined as left bundle branch block, or right bundle branch block plus left anterior or posterior fascicular block. Patients signed a written informed consent. The study protocol conforms to the ethical guidelines of the 1975 Declaration of Helsinki as reflected in *a priori* approval by the institution’s human research committee (The Clinical Research Ethics Committee of Granada (CEIC). Patients were excluded from the study in case of structural heart disease, defined as interventricular septal wall thickness >13 mm, left ventricular ejection fraction <50%, valvular heart disease ≥ grade 2, or ECG and cardiac imaging findings suggestive of arrhythmogenic cardiomyopathy^5^ (Patients who met diagnostic criteria for arrhythmogenic dysplasia of the right ventricle, two major criteria, one major and two minor or four minor were excluded). Patients were also excluded if they fulfilled ECG criteria for a cardiac channelopathy^6,7^ or in the presence of family history of early sudden cardiac death.

Patients were classified in two groups according to the initial therapeutical approach at the physician’s criteria: one group received a direct pacemaker implant, while the remaining were treated with a pacemaker basing upon the EPS result according to current guidelines^4^.

Briefly, EPS was performed following a standardised protocol as follows: sinus and atrioventricular node functions were analysed at the basal point and under the effect of class IC drugs. The EPS was considered abnormal in case of a baseline HV interval ≥70 ms or in the presence second- or third-degree His–Purkinje AV block during incremental atrial pacing or after intravenous class IC antiarrhythmic drugs^8^. If negative, an ILR was placed. The implantable loop recorder findings were considered diagnostic in case of Sinus bradycardia with pauses greater than 3 seconds, sustained ventricular tachycardia, Mobitz II or III degree AV block.

### Endpoints

Clinically relevant events during follow-up were analysed in both groups, including syncope recurrence, death from any cause, complications of pacemaker, EPS or implantable loop recorder which requires hospitalization greater than 24 hours or intervention for resolution, symptomatic AV block requiring medical attention and a combined endpoint of all of these variables. For all variables except the combined endpoint, only the first event was considered. Other secondary variables such as ventricular arrhythmias observed either in the ILR or the pacemaker, falls and fractures secondary to syncopal attacks, as well as percentage of ventricular stimulation in pacemakers and progression to complete AV block after one year, were collected.

### Statistical analysis

The continuous variables are expressed as mean and standard deviations. The qualitative variables are expressed as absolute numbers and percentages. The Student’s t-test was used to compare two means, and analysis of variance (ANOVA) and the Newman-Keuls method for multiple comparisons. The Chi-square test was used to compare proportions. Multiple logistic regression was used for the multivariate analysis using the stepwise method to select variables. P-values < 0.05 were considered statistically significant.

The Kaplan-Meier estimator was used to evaluate the occurrence over time of the different events, analysed as censored events. The differences between the two treatment groups were studied using the log-rank test. Hazard ratios (HR) were calculated using Cox regression.

## Results

**Figure 1:**
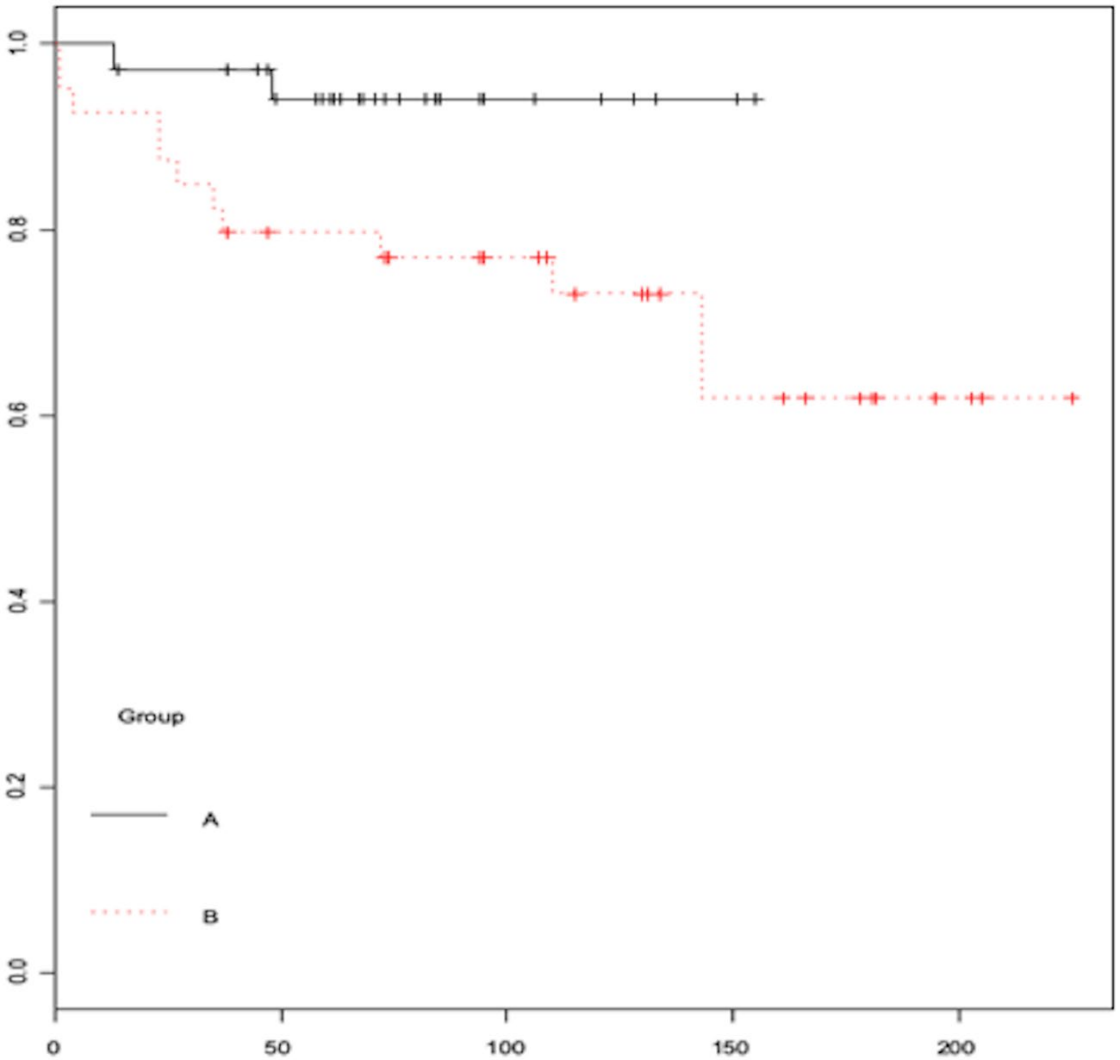
Time to event curves for syncope comparing groups A and B.

**Figure 2:**
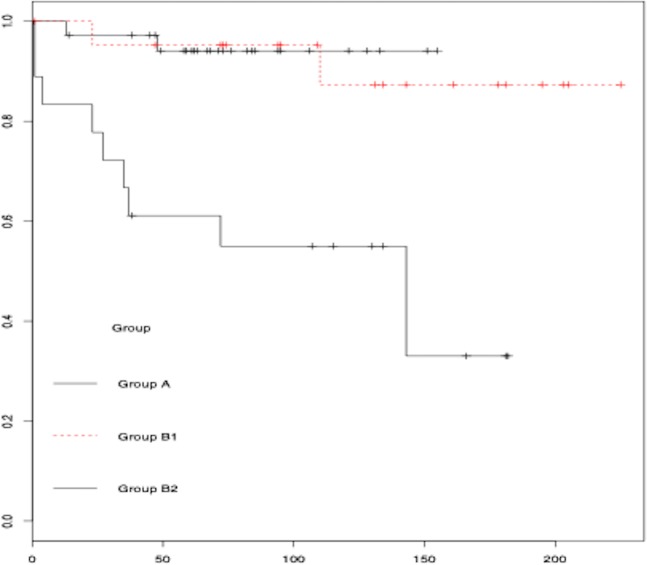
Time to event curves for syncope comparing groups A, B1 and B2.

Seventy seven patients with BBF and syncope were included in the study (61% male, aged 71.7 ± 9.6 years), 36 of them were patients treated directly with an empirical pacemaker (Group A) and 41 with a clinical decision based on the results of the EP study (Group B). Among these, 23 patients received a pacemaker after a positive EPS, and 18 received an ILR after a negative EPS. Table 1 shows the baseline characteristics of the two groups. The mean follow-up: 81.6 ± 33.2 in Group A and 126.4 ± 56.3 in Group B. During a mean follow-up period of 105.4 ± 51 months, 18% of all patients had at least one syncopal episode. Theses recurrences were less common in Group A than Group B (Table 1, Fig. 1; 5.6% vs. 29.3%; p = 0.02). In Group B, the incidence of syncope was lower in patients who received a pacemaker than in those who received an ILR (Table 2, Fig. 2; 9.3% vs. 55.6%; p = 0.02). Among all patients who finally received a pacemaker, there were no differences in terms of syncope during follow-up. Independent predictors of syncope recurrence were the treatment approach based on EPS results, a medical history of diabetes mellitus and the presence of left bundle branch block (LBBB) (Table 3).

**Table 1:** Patient characteristics and events.

	Total	Empirical PM	EPS	P
	(N = 77)	(N = 36)	(N = 41)	
Age (years)	71.74 ± 9.6	76.2 ± 7.6	67.7 ± 9.5	0.01
DM	15(19.5%)	10(27.8%)	5(12.2%)	0.08
HTN	42(54.5%)	21(58.3%)	21(51.2%)	0.53
LBBB	32(41.6%)	12(33.3%)	20(48.7%)	0.17
QRS (ms)	142.8 ± 18.3	137.3 ± 17.8	147.7 ± 17.7	0.01
PR (ms)	198 ± 45.1	208.4 ± 57.5	189.5 ± 29.4	0.07
VP	53.8 ± 40.8	49.4 ± 41.4	58.6 ± 40.3	0.35
AP	16.4 ± 11.4	17.9 ± 13.6	15.0 ± 9.2	0.39
Progression to AVB	24(33.8%)	11(30.6%)	13 (39.4%)	0.44
Sex (male)	47(61%)	25(69.4%)	22(53.6%)	0.15
AF	9(11.7%)	6(16.7%)	3(7.3%)	0.29
ICD	0	0	0	
Clinical VT	0	0	0	
Subclinical VT	1 (1.3%)	1 (2.78%)	0	0.85
Syncope	14 (18.2%)	2 (5.6%)	12 (29.3%)	0.02
Complications	19 (24.7%)	8 (22%)	11 (26.8%)	0.89
Death	25 (32.5%)	12 (33.3%)	13 (31.7%)	0.97
Bradycardia	13 (17.2%)	1 (2.8%)	12 (29.3%)	0.02
Compound	1.01 ± 1.21	0.64 ± 0.62	1.34 ± 1.44	0.01

PM: pacemaker; EPS: electrophysiology study; DM: diabetes mellitus; HTN: hypertension; LBBB: left bundle branch block; QRS: QRS interval; PR: PR interval; VP: Ventricular pacing; AP: Atrial pacing; AVB: atrioventricular block AF: atrial fibrillation; ICD: implantable cardioverter-defibrillator; VT: ventricular tachycardia.

X^2^ was used for the qualitative variables.

**Table 2:** Baseline characteristics and events by post-EPS treatment approach.

	Total	Empirical PM	EPS + PM	EPS−PM	p
	(N = 77)	(N = 36)	(N = 23)	(N = 18)	
Age (years)*	71.7 ± 9.6	76.2 ± 7.6	67.7 ± 8.7	67.8 ± 10.8	0.01
QRS (ms)	142.8 ± 18.3	148.7 ± 17.0	145.8 ± 19.1	137.3 ± 17.8	0.05
PR (ms)	198.0 ± 45.1	208.4 ± 57.5	196.5 ± 32.1	180.4 ± 24.6	0.11
Progressión to AVB	24(33.8%)	11(30.6%)	8(34.8%)	5(41.7%)	0.77
VP	53.8 ± 40.8	49.4 ± 41.4	57.4 ± 40.4	60.7 ± 41.9	0.63
AP	16.4 ± 11.4	17.9 ± 13.6	16.3 ± 8.9	12.4 ± 9.7	0.48
SNRTc				330 ± 118	
Sex (male)	47(61%)	25(69.4%)	13(56.5%)	9(50%)	0.35
DM	15(19.4%)	10(27.7%)	3(13.4%)	2(11.1%)	0.22
HTN	42(54.5%)	21(58.3%)	13(56.5%)	8(44.4%)	0.65
LBBB	32(41.6%)	12(33.3%)	9(39.1%)	11(61.1%)	0.14
AF	9(11.7%)	6(16.7%)	1(4.3%)	2(11.1%)	0.35
Syncope	14 (18.2%)	2 (5.6%)	2 (9.3%)	10 (55.6%)	0.01
Complications	19 (24.7%)	8 (22%)	8 (34.8%)	3 (16.67%)	0.19
Death	25 (32.4%)	12 (33.3%)	6 (26%)	7 (38.9%)	0.41
Bradycardia	13 (16.8%)	1 (2.8%)	1 (4.35%)	11 (61.1%)	0.01
Compound*	1.01 ± 1.21	0.64 ± 0.62	0.78 ± 0.95	2.05 ± 1.66	0.01

*Differences between group B2 and all groups (Newman-Keuls test); PM: pacemaker; EPS: electrophysiology study; QRS: QRS interval; PR: PR interval; AVB: Atrioventricular block; VP: Ventricular pacing; AP: Atrial pacing; SNRTc: Corrected sinus node recovery time; DM: diabetes mellitus; HTN: hypertension; LBBB: left bundle branch block; AF: atrial fibrillation. X^2^ was used for the qualitative variables.

**Table 3:** Multivariate analysis and Cox regression.

Variable	HR	P
**Model 1. Dependent variable: syncope**		
EPS vs. PM	6.24 (1.26–30.97)	0.025
DM	4.13 (1.15–15.79)	0.029
LBBB	3.62 (1.15–14.79)	0.033
**Model 2. Dependent variable: bradycardia**		
EPS vs. PM	8.01 (1.026–63.94)	0.047
**Model 3. Dependent variable: mortality**		
EPS vs. PM	0.67(0.24–1.57)	0.28
Age	1.10 (1.03–1.17)	0.01
HTN	2.68 (1.06–6.79)	0.03

PM: pacemaker; EPS: electrophysiology study; DM: diabetes mellitus; HTN: hypertension.

Syncope recurrence at 40 months was lower in Group A (2.7% (1) vs. 19.5% (8), p = 0.03).

Among the 41 patients who underwent an EPS, 35 received a pacemaker (85%), 23 of them at the first time because the EPS showed an abnormal AV conduction. Of the 18 who did not receive a pacemaker immediately after the EPS, 12 received it later mainly due to progression to complete/advanced AV block.

Regarding the occurrence of symptomatic AV block during follow-up, it occurred in 13 patients among the whole sample (17.1%). As Table 1 shows, AV block was more common in Group B than A (2.8% vs 29.3%, p = 0.022), particularly due to a high incidence in patients who were treated with an ILR and did not receive a pacemaker initially (61.1%) (Table 2). Only 1 traumatic injury associated with bradycardia occurred in Group A (2.8%), compared with 4 in Group B (9.7%, p = 0.36).

### Complications (Table 1, and 2)

Complications occurred in 19 patients (24.7%). There were no differences between the groups (A 22%; B1 34.8%; B2 16.7%).

### Mortality (Tables 1, 2 and 3)

Twenty five patients (32.5%) died from any cause during follow-up and 7 (9.1%) were lost to follow-up. In group A, 12 patients (33.3%) died from any cause and 3 were lost to follow-up (8.3%), while in group B, 13 died from any cause (31.7%) and 4 were lost to follow-up (9.8%) (p = 0.97) (Table 1). Only age and hypertension were retained as independent predictor of mortality (Table 3).

Total number of events (Tables 1, 2, 4, and Table 5).

**Table 4:** Multivariate analysis by multiple linear regression. Dependent variable: number of events (syncope, AV block, complications of procedure or death).

Variable	Beta coefficient	Standard error B	p
EPS vs. PM	0.703	0.266	0.01
**R** = **0.29**			

PM: pacemaker; EPS: electrophysiology study.

**Table 5:** Distribution of number of events (syncope, AV block, complications of procedure or death) by strategy used.

	Total	Empirical PM	EPS + PM	EPS−PM	p
	(N = 77)	(N = 36)	(N = 23)	(N = 18)	
Number of events					0.007
0 events	40.3%	47.2%	47.8%	16.7%	
1 event	35.1%	44.4%	34.7%	16.7%	
>2 events	24.7%	8.3%	17.4%	66.7%	

PM: pacemaker; EPS: electrophysiology study.

The total number of events during follow-up (syncope, symptomatic AV block, complications of the procedure or death) was lower in Group A (0.64 ± 0.62) than in Group B (1.34 ± 1.44) (p = 0.010). When grouping the number of events in intervals, events were more common in group B with a statistically significant difference. Table 5 shows the distribution of events in groups A and B. These differences were even more relevant when all three groups were analysed. The number of events in patients who only received an ILR was 2.05 ± 1.66 (p < 0.001 by ANOVA), and the Newman-Keuls test showed differences between this group and the others, but no statistically significant differences between groups A and B was found (Tables 2, 5).

## Discussion

Management of patients with unexplained syncope and BFB has remained controversial throughout the years. According to our work, an approach consisting of a direct pacemaker implant significantly decreases the risk of syncope recurrence and progression to complete or advanced AV block in a prospective follow-up, respect to those treated with a conservative approach based on the results of an EP study. Indeed, the incidence of syncope in patients who received a pacemaker after a positive EPS was similar to the one observed in patients empirically treated. These events did not mean a survival improvement in those treated with a pacemaker, likely due to a high mortality rates observed in a very old sample population.

Some studies have analysed the clinical involvement of BFB^9–11^ and its relation to syncope of unknown origin^3,12–16^. According to the latest pacing clinical practice guidelines, and summarizing available evidence, syncope in patients with BFB and an abnormal EPS should receive a pacemaker. If the results of the EPS are inconclusive, implantation of an ILR is recommended. A direct pacemaker implantation is suggested only as a class IIb recommendation (level of evidence B) if the diagnostic tests are inconclusive.

However, the sensitivity of EPS to detect patients at risk of AV block during follow-up is low^4^. In a recent work by Roca-Luque *et al*.^17^, almost 25% of patients with negative EPS developed advanced AV block during a 25 month follow-up period, requiring, thus, a pacemaker. Interestingly, this proportion seemed to be lower when the EPS was performed under flecainide than under procainamide. In our study, 55% of patients with negative EPS results had a syncopal recurrence during follow-up. Paroxysmal AV block was recorded in a similar rate in the ILR group. One of the relevant medical consequences of these recurrences might be a higher incidence of severe traumatic injuries, something that we could not demonstrate in our study due to, likely, the sample size. Similarly, syncope recurred in a 33% of patients who received event monitors in the B4 study^2^. These data, taken together with the high incidence pacemaker implantation during follow-up in patients who underwent an EPS (85% in our serie), raise the question of a more cost-effective approach of the direct pacemaker implant^18^.

On the other hand, in the PRESS study^3^, the incidence of syncope in patients with pacemakers programmed in DDD mode at 60 bpm was similar (13%) to that in patients with pacemakers with a DDI mode at 30 bpm (control group). In both groups the baseline EPS result was negative. However, the incidence of presyncope associated with bradycardia and symptomatic AV block were significantly higher in the control group. Taking these data into consideration, an empirical pacemaker implantation might be a reasonable therapeutic option despite syncope recurrence in some patients. Notably, in that study no ventricular arrhythmias were recorded in patients with syncope and presyncope recurrence.

Kalscheur *et al*.^19^ reported a 27% incidence of syncope recurrence in patients who received empirical pacing. These patients were compared with those who received a pacemaker after a positive EPS or after AV block was documented by the ILR. There were no syncope recurrences in the second group. Interestingly, the authors did not report the incidence of syncope recurrence in patients with a negative EPS nor which group the patients with events recorders came from. The syncope recurrence rate in the empirical pacemaker group was quite higher than the one reported in previous studies (13%^3^ and 7%^20^). It was even substantially higher than in the B4 study^2^ where the 19-month recurrence rate was 15%, despite almost a third of patients in that study not having pacemakers, making them the ones most at risk of events. These all suggest, as the authors themselves state, that empirical pacing tends to be used in frailer, more elderly patients where other causes of syncope may be more relevant.

One of the main drawbacks of empirical pacemaker implantation in syncope patients is that ventricular tachycardia might be the aetiology. However, ventricular tachycardia induction in structurally normal hearts is extremely rare^21^. Ventricular tachycardia induction during EPS in patients with syncope of unknown origin and BFB occurs almost exclusively in patients with structural heart disease^22^. In fact, EPS has been performed without any ventricular tachycardia induction at all in groups of more than 200 patients with BFB without structural heart disease^20^. In our study, no specific ventricular arrhythmias were induced in the 41 EPS performed, no symptomatic or asymptomatic ventricular tachycardias were recorded, no ICD implantation was required during follow-up, and none of the deceased patients suffered a sudden cardiac death. Although an insertable Holter monitor would help clarify which patients need a pacemaker, this usually involves a new syncopal attack, and this might have serious consequences, especially in elderly patients^23^. Furthermore, the cost of this strategy is evident^17,24^, given the fact that 86% of patients in our study finally needed a pacemaker. This is consistent with the findings of previous studies^2^ where pacemakers were implanted in 70% of patients with syncope of unknown origin and BFB. However, randomized studies^14^ to clarify the role of events recorders in the management of these patients are currently ongoing.

## Limitations

As this is an observational study, the strategy in each case was selected by the clinical cardiologist. However, based on our initial experience, gradually empirical pacing became the most frequent treatment approach.

Although the baseline characteristics of the groups were not the same, they were more unfavourable in the empirical treatment group, primarily because of patient age. Nevertheless, this group had a clear clinical benefit compared with the EPS group. The low rate of syncopes after pacemaker implantation suggests that bradycardia is the most common cause of syncope.

Although the population studied was small, because the condition studied has a high rate of recurrence and events, and a long follow-up period was used, statistical significance was achieved.

## Conclusions

Most patients with syncope and BFB develop advanced AV block and/or syncope during follow-up. Our study shows treatment according to EPS does not improve the results of another treatment strategy with empirical pacemaker. Randomized studies are necessary to confirm our results.
